# Comprehensive analysis of the association of seasonal variability with maternal and neonatal nutrition in lowland Nepal

**DOI:** 10.1017/S1368980021003633

**Published:** 2022-07

**Authors:** Naomi M Saville, Mario Cortina-Borja, Bianca L De Stavola, Emma Pomeroy, Akanksha Marphatia, Alice Reid, Dharma S Manandhar, Jonathan CK Wells

**Affiliations:** 1 Institute for Global Health (IGH), University College London (UCL), London, UK; 2 Great Ormond Street Institute of Child Health (ICH), University College London (UCL), London, UK; 3 Department of Archaeology, University of Cambridge, Cambridge, UK; 4 Department of Geography, University of Cambridge, Cambridge, UK; 5 Mother and Infant Research Activities (MIRA), Kathmandu, Nepal

**Keywords:** Seasonality, Cosinor models, Nepal, Newborn anthropometry, Nutrition in pregnancy

## Abstract

**Objective:**

To provide a comprehensive seasonal analysis of pregnant mothers’ eating behaviour and maternal/newborn nutritional status in an undernourished population from lowland rural Nepal, where weather patterns, agricultural labour, food availability and disease prevalence vary seasonally.

**Design:**

Secondary analysis of cluster-randomised Low Birth Weight South Asia Trial data, applying cosinor analysis to predict seasonal patterns.

**Outcomes:**

Maternal mid-upper arm circumference (MUAC), BMI, dietary diversity, meals per day, eating down and food aversion in pregnancy (≥31 weeks’ gestation) and neonatal z-scores of length-for-age (LAZ), weight-for-age (WAZ) and head circumference-for-age (HCAZ) and weight-for-length (WLZ).

**Setting:**

Rural areas of Dhanusha and Mahottari districts in plains of Nepal.

**Participants:**

2831 mothers aged 13–50 and 3330 neonates.

**Results:**

We found seasonal patterns in newborn anthropometry and pregnant mothers’ anthropometry, meal frequency, dietary diversity, food aversion and eating down. Seasonality in intake varied by food group. Offspring anthropometry broadly tracked mothers’. Annual amplitudes in mothers’ MUAC and BMI were 0·27 kg/m^2^ and 0·22 cm, with peaks post-harvest and nadirs in October when food insecurity peaked. Annual LAZ, WAZ and WLZ amplitudes were 0·125, 0·159 and 0·411 z-scores, respectively. Neonates were the shortest but least thin (higher WLZ) in winter (December/January). In the hot season, WLZ was the lowest (May/June) while LAZ was the highest (March and August). HCAZ did not vary significantly. Food aversion and eating down peaked pre-monsoon (April/May).

**Conclusions:**

Our analyses revealed complex seasonal patterns in maternal nutrition and neonatal size. Seasonality should be accounted for when designing and evaluating public heath nutrition interventions.

Highly seasonal environments impose multiple stresses that may impact various aspects of human nutrition, including dietary intake, physical activity patterns and somatic nutritional status^(1–3)^. Seasonal effects during early development, especially in the first 1000 d, may have long-term consequences^(4)^. Seasonal effects have previously been reported in maternal nutrition and birth weight^(5–8)^, neonatal survival^(8)^ and physical activity and dietary intakes of pregnant mothers^(9)^.

In the plains of South Asia, weather patterns, agricultural labour activities, food availability and disease prevalence all vary greatly by season. During the pre-monsoon/monsoon seasons (April–September), high temperatures and humidity cause heat stress^(10)^, associated with lower birth weight^(11)^ and increased prevalence of infectious diseases^(10,12–15)^. Conversely, low temperatures stimulate energy expenditure (heat production)^(16)^, but are also associated with increased neonatal mortality from hypothermia^(17)^, higher prevalence of acute respiratory infections^(18,19)^ and higher indoor air pollution from cooking and heating^(20,21)^, which may impact negatively upon birth weight^(22)^.

Agricultural cycles are associated with seasonal food shortages^(13)^ that may particularly impact women in South Asia, due to their large burden of agricultural work^(23–25)^ and their responsibility for preparing family meals^(26)^. During periods of peak agricultural work, pregnant women may experience both high energy expenditure and restricted food intake, due to lack of time to manage meals^(27)^, while seasonal availability affects intake of micronutrient-rich foods^(28)^. Intra-household allocation of food may become more inequitable during seasonal food shortages^(29)^, which adversely affect women due to their lower household status^(30)^.

Whilst seasonal patterns of food security, energy expenditure and disease have been described in Nepal, previous studies have generally included only limited nutritional outcomes, resulting in a fragmentary overview. This study provides a comprehensive seasonal analysis of maternal and newborn nutrition in an undernourished population from the plains (*Terai*) to better understand the timing of nutritional vulnerability in mothers and their newborns, and inform interventions aimed at tackling undernutrition in pregnancy and infancy. We model seasonal patterns in somatic outcomes, pregnancy diet quality/quantity and eating behaviour using cosinor analysis.

## Methods

### Study setting and participants

We analysed data from a cluster-randomised controlled trial conducted with Maithili-speaking women in Nepal’s plains, where low maternal BMI (<18·5 kg/m^2^) and anaemia and child wasting are common, and women’s agency is poor^(31)^. Supplemental Figure S1 illustrates monthly patterns of weather, agricultural activities and the lean season in our study area. There is substantial variability in temperature, humidity and precipitation, exerting contrasting composite ecological stresses by season.

The protocol, methods and results of the cluster-randomised controlled trial have been reported^(32–36)^. Briefly, the four-arm trial tested the impact of community interventions in pregnancy upon birth weight and infant weight-for-age z-score (WAZ) at the trial endpoint. The unit of randomisation was a ‘Village Development Committee’ geopolitical administrative area. All pregnant women in each cluster could enrol at any gestational age. Interventions tested involved participatory learning and action (PLA) women’s groups focussed upon nutrition in pregnancy, with and without transfers of food or cash to pregnant mothers. Whilst birth weight increased in PLA only, PLA + cash and PLA + food arms compared with the control arm, this was only significant in the PLA + food arm. No anthropometric differences were found at endpoint, when child age averaged 9 months.

The trial enrolled 25 090 women. All data were collected between January 2014 and April 2015 by local interviewers, educated mostly to bachelors’ level and given several weeks of training on interviewing, dietary assessment and anthropometry. Varying numbers were captured at different time points (Fig S2). Infants measured >3 d of birth were excluded, resulting in 3330 measures ≤72 h of birth and 2846 women in pregnancy. Loss to follow-up at different time points was due to data collector overload (more women enrolled than predicted), political unrest affecting data collector performance, women migrating to their parental home in late pregnancy for delivery and halting of funding before all outcomes could be collected. Technical error of measurement and coefficient of reliability for intra- and inter-observer variability was evaluated after each training session, and data collectors were retrained as needed.

### Data collection

Maternal weight, height and mid-upper arm circumference (MUAC) after 31 weeks’ gestation and neonatal weight, length and head circumference (HC) were measured using: Tanita BD590 scales (infants, precision 10 g); Tanita solar scales (mothers, precision 100 g); ‘Shorr boards’ for length/height (precision 1 mm) and Seca 212 circumference tapes for MUAC and head circumference. We calculated anthropometric z-scores for WAZ, length-for-age (LAZ), and head circumference-for-age (HCAZ) and weight-for-length (WLZ) using WHO 2006 growth standards and cleaning criteria and maternal BMI^(37)^. Measures were obtained across the year but with a gap from 20 May to 7 July 2014 inclusive, when training prevented data collection (Supplemental Fig S3). Whilst this gap spanned the late pre-monsoon and early monsoon, there were sufficient measures before and after the gap for these seasons to be well represented in the data.

During pregnancy (≥31 weeks), we asked mothers to recall intake of ten food groups in the previous 24 h using the standard FAO Women’s Dietary Diversity Score (WDDS) questionnaire^(38)^, which was newly released at the time of the study. This tool has been shown to be a good proxy for mean probability of adequacy of diets in Zambia^(39)^ and amongst pregnant women in Bangladesh^(40)^. We also asked at ≥31 weeks: (i) if they had been eating less, more or the same amount in the last 2 weeks compared to pre-pregnancy; (ii) if they suffered severe loss of appetite or food aversion after the 4th month; (iii) if they had vomited in the last 2 weeks; (iv) how many main meals and snacks they had consumed in the preceding 24 h. At the early pregnancy interview (<31 weeks), we used the Months of Adequate Household Food Provisioning questions to estimate food security preceding and during early pregnancy^(41)^. For those who reported food insecurity, we asked which Nepali months over the last year had shortage of food.

### Statistical methods – modelling of seasonality

We fitted cosinor regression models to detect seasonality in annual and half-yearly (semestral) cycles and to describe the peak and nadir of selected outcomes^(42–45)^. To avoid confusion arising from discrepancies in the definition of Nepalese seasons and use of Gregorian/Nepalese calendars, we analysed day in the year as a continuous variable.

Cosinor linear regression models for each maternal and newborn outcome were specified with annual and semestral cycles, to fully capture their seasonality. To maximise comparability across outcomes, we fitted both terms in all models.

We first create a day in the year variable, 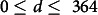 for date of birth or measurement and transform it to angles:

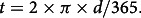




For simplicity, we define below the model for an annual cycle only. The full model specified with annual and semestral cycles and the model specification for binary outcomes are provided in Annexe S5.

An annual cycle model for a continuous outcome 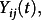 measured in mother (or child) *i* belonging to cluster *j* for angle *t* (in radians, corresponding to transformed day of the year), is specified as a mixed-effects model:

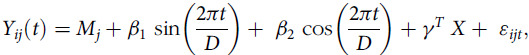

where 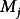 = 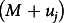 is the random coefficient representing the average outcome (mesor) for cluster *j*, with 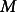 the overall average while 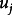 is a random effects term assumed to be normally distributed with mean 0 and variance 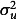 ; sin(.) and cos(.) are trigonometric functions of *t* (with 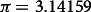 and *D* = 365, the total number of days in a year); 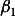 and 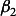 are the regression coefficients for the annual cycle and 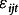 is a residual term, assumed to be normally distributed independently of 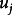 with mean 0 and variance 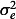 . 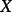 is a design matrix of covariates for parameters 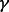 .

This model is equivalent to the following linear regression representation:

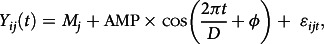

where:

AMP = Amplitude = 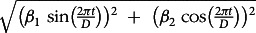


and

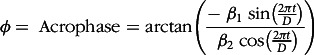




The acrophase 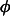 can be converted into days (from the start of the cycle) as follows:

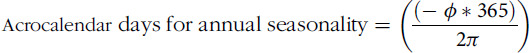




The numerical day in the year can then be converted back into a calendar day (e.g. 100 is 10th April in a non-leap year).

**Fig. 1:**
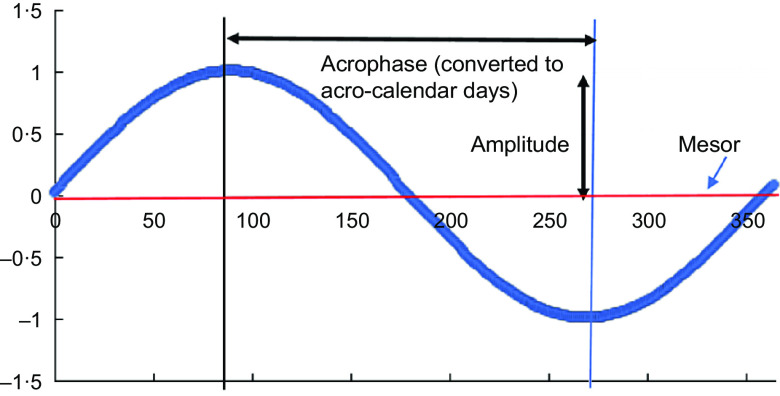
Schematic diagram of cosinor analysis output

Figure 1 illustrates the role played by mesor, amplitude and acrophase in the cosinor model for a particular cluster and shows how with this model there is one peak and one nadir in the values of 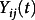 per year. Semestral cosinor terms (specifically, 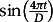 and 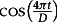 where *D* = 182·5) allow for seasonal cycles with two peaks and two nadirs per year. Models were adjusted for age of the infant, gestational age in weeks, primigravid status of the mother and trial study arm.

These models provided: (i) coefficients for the sine and cosine functions that were then used to derive predicted seasonal patterns; (ii) yearly and semestral amplitudes describing the magnitude of seasonal variability measured from the mid-point in the cluster-specific mesor (presented in the units of measurement for continuous outcomes and in probabilities for binary outcomes (with CI derived from bootstrapped standard errors with 1000 samples)); (iii) yearly and semestral acrophases measuring distance from the start of the cycle (1 Jan) to its peak/nadir, which we converted into calendar days of the high/low points (with bootstrapped CI).

The *P*-values for pairs of annual, and pairs of semestral, sine and cosine functions are presented separately to enable the relative importance of annual and semestral cycles to be compared across outcomes.

We predicted seasonal patterns and their 95 % CI for each outcome by combining the relevant estimated parameters and their variance–covariance matrices and plotted them using the ggplot2 package in R^(46)^. For continuous variables, plots are presented in the units of measurement whereas plots for binary variables are presented as predicted probabilities of a positive recall for that outcome against day in the year. The scale of the *y*-axes is expanded to enable easy detection of patterns of predicted seasonal change and comparisons between plots.

### Statistical methods – of covariates and outcomes

Our primary objective was to test for seasonality in dietary indicators in pregnancy and anthropometric outcomes in pregnancy and at birth, hence we did not build causal models. Before fitting cosinor regression models on our outcomes of interest, we tested for seasonality in the following variables to investigate whether they might confound the association between seasonality and the outcomes, by fitting regression models with a cosinor term: maternal height, maternal age, gestational age, primigravida status and infant age. We found significant seasonality in primigravida, infant age and gestational age, but not in maternal age or height. In Nepal, where childbearing is very rare outside of marriage, seasonality in primigravidae may be due to marriage seasonality whereby certain months are considered auspicious for marriage. Seasonality in infant and gestational ages may reflect differing timing of interviews. To reduce noise from these factors, we adjusted neonatal outcomes for log-transformed infant age (days), maternal outcomes for gestational age (weeks) and all outcomes for primigravid status. Model intercepts varied by the unit of randomisation to account for clustering of the outcomes within each unit (*M*_*j*_ in the equations). Since trial interventions could potentially have influenced seasonal patterns, we also adjusted for study arm. Hence, each plot of predicted seasonal patterns for each outcome represents the reference values of multigravida and control study arm, and average gestational age (in maternal models) or child age (in newborn models).

Continuous outcomes included mothers’ MUAC, BMI, WDDS and number of main meals and snacks per day (meals) at ≥31 weeks’ gestation, and neonatal HAZ, WAZ, WLZ and HCAZ (≤72 h from birth) by day in the year. To get an indication of the peak and nadir values of continuous variables, it is possible to add or subtract the amplitude from the mesor. Binary outcomes, analysed with mixed-effects logistic regression cosinor models, were eating less than when not pregnant (eating down), vomiting, diarrhoea in the last 2 weeks, aversion to food/lack of appetite and consumption of each of nine individual food groups (meat/fish, dairy, eggs, pulses, nuts and seeds, green leafy vegetables (GLV), vitamin A-rich fruits and vegetables, other vegetables and other fruits) in the preceding 24 h, all measured ≥31weeks’ pregnancy.

Analyses were performed in Stata 15·1 (StataCorp LLC, College Station, Texas) and R 4·0·2 (The R Foundation for Statistical Computing, Vienna, Austria).

## Results

### Respondent characteristics

Table 1 contrasts respondent characteristics for women sampled at pregnancy and/or birth *v*. women not followed-up at these time points. Due to implementation challenges, such as political unrest and cessation of funding, only a small proportion of the 25 090 pregnancies enrolled were captured at ≥31 weeks’ gestation and/or within 3 d of birth. However, differences between captured and not captured groups were small (0–3 percentage points), so we deem the pregnancy and delivery samples representative of those enrolled in the study.

**Table 1:** Respondent characteristics

	N	Not sampled in late pregnancy		Sampled in late pregnancy		Not sampled in delivery questionnaire		Sampled in delivery questionnaire		Enrolled in LBWSAT overall	
**Age of Mother**	25 054										
9–14 years*		119	1 %	17	1 %	116	1 %	20	1 %	136	1 %
15–16 years		1896	9 %	216	8 %	1829	8 %	283	8 %	2112	8 %
17–18 years		4332	20 %	538	19 %	4281	20 %	589	18 %	4870	19 %
19 years		2852	13 %	367	13 %	2801	13 %	418	13 %	3219	13 %
20–24 years		8262	37 %	1066	38 %	8083	37 %	1245	37 %	9328	37 %
25–29 years		3586	16 %	468	16 %	3481	16 %	573	17 %	4054	16 %
30–51 years		1167	5 %	168	6 %	1133	5 %	202	6 %	1335	5 %
9–14 years		119	1 %	17	1 %	116	1 %	20	1 %	136	1 %
**Mothers’ education**	24 162										
Never went to school		13 667	64 %	1821	64 %	13 290	64 %	2198	66 %	15 488	64 %
Primary to lower secondary		4341	20 %	575	20 %	4233	20 %	683	21 %	4916	20 %
Secondary and above		3314	16 %	444	16 %	3309	16 %	449	13 %	3758	16 %
**Farmer** (produces food from own or share-cropped land or labour exchange)	21 098	15 898	87 %	2406	86 %	15 529	87 %	2775	85 %	18 304	87 %
**Study arm woman enrolled in**	25 090										
Control		4681	21 %	628	22 %	4621	21 %	688	21 %	5309	21 %
Women’s group		5091	23 %	535	19 %	4917	23 %	709	21 %	5626	22 %
Cash		6362	29 %	909	32 %	6416	29 %	855	26 %	7271	29 %
Food		6116	27 %	768	27 %	5806	27 %	1078	32 %	6884	27 %
**First pregnancy is this index pregnancy**	24 144	7652	36 %	989	35 %	7532	36 %	1109	33 %	8641	36 %
**Sex of infant**	19 167										
Boy		8798	53 %	1336	51 %	8421	53 %	1713	53 %	10 134	53 %
Girl		7735	47 %	1298	49 %	7503	47 %	1530	47 %	9033	47 %
		**Mean**	**sd**	**Mean**	**sd**	**Mean**	**sd**	**Mean**	**sd**	**Mean**	**sd**
**Weeks of gestation at pregnancy interview** (mean, sd)	2884	36·05	3·01	35·64	2·79					35·65	2·79
**Weeks of gestation at delivery** (mean, sd)	19 695					40·00	9·27	39·93	3·95	39·99	8·60
**Newborn age in days at delivery interview** (mean, sd)	6587					17·48	14·91	1·20	0·75	9·25	13·28
**Total**		**22 250**	**100 %**	**2840**	**100 %**	**21 760**	**100 %**	**3330**	**100 %**	**25 090**	**100 %**

* Minimum age was 12 years in the sample analysed but there were only 1–3 individuals in the 12- and 13-year age categories. In the group that was not sampled at either time point, there was one mother each in the age categories of 9, 10 and 11 years old. It was not possible to verify the validity of these young maternal ages since respondents usually are unable to recall their date or birth.

The population comprises 84 % Hindu and 16 % Muslim, Maithili-speaking *Terai* castes, including 21 % marginalised Dalit groups and very few women of hills ethnicity. Three quarters of households had no toilet and ˜95 % cooked on a mixture of dried dung, firewood and agricultural residues that elevate indoor pollution. Most (86 %) belonged to farming families that sourced staple foods via production from their own or share-cropped land or through agricultural labour exchange. Most households purchase locally grown food (especially fruits and vegetables) from the local ‘haat bazaar’ market.

Landholding sizes were small, half a hectare on average. Over one-third of the pregnant mothers were primigravid. Most were adolescent (40 %) or in their early twenties (mean age 21·7 years, sd 4·7) with 8 % aged 15–16 years and just 1–2 and 2–3 individual mothers aged 12 and 13 years, respectively, in the analysed samples. The majority (89 %) had married below 18 years (mean 15·2 years, sd 2·1). Two-thirds of women had no schooling and 20 % had primary education only, while husbands had only slightly better educational opportunities. Mean gestational age at sampling in pregnancy was 35·6 weeks. After exclusion of newborns measured >72 h, mean age at sampling was 1·2 d. Boys comprised 53 % of the sample.

Supplemental Figure S4 shows recalled Nepalese month of food insecurity amongst 217 mothers who experienced months of food shortage in the year before the early pregnancy interview (recalling the period 27 Jan 2013–31 March 2014). The main ‘lean period’ falls in the post-monsoon months of mid-September to mid-November, just preceding rice harvest. Food insecurity falls to the lowest prevalence during the rice harvest in mid-November to mid-December and remains uncommon while rice stocks last until mid-February. From then (beginning of the hot pre-monsoon) until August (monsoon), food insecurity remains moderate with a slight improvement around pulse and wheat crop harvest in March/April. These food stocks remain up to the end of the monsoon at which point food insecurity peaks sharply.

### Cosinor analyses

**Fig. 2:**
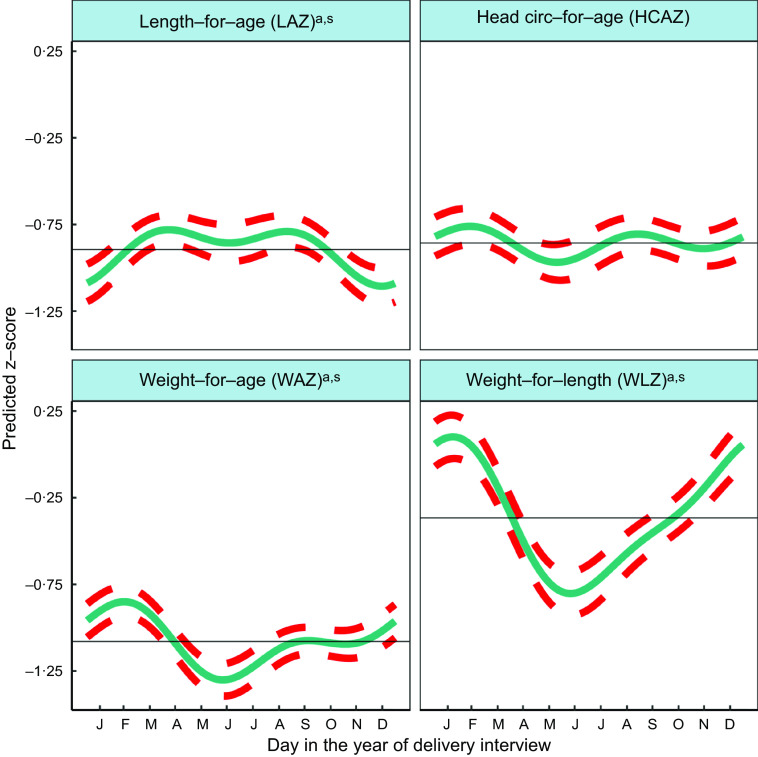
Predicted seasonal patterns of neonatal anthropometric z-scores for length-for-age (LAZ), weight-for-age (WAZ), weight-for-length (WLZ) and head circumference-for-age (HCAZ) at birth plotted using cosinor analysis outputs analysed in relation to day of birth. a = significant annual cycle; s = significant semestral cycle; LAZ *n* 3291, WAZ *n* 3330; WLZ *n* 3029, HCAZ *n* 3200

**Fig. 3:**
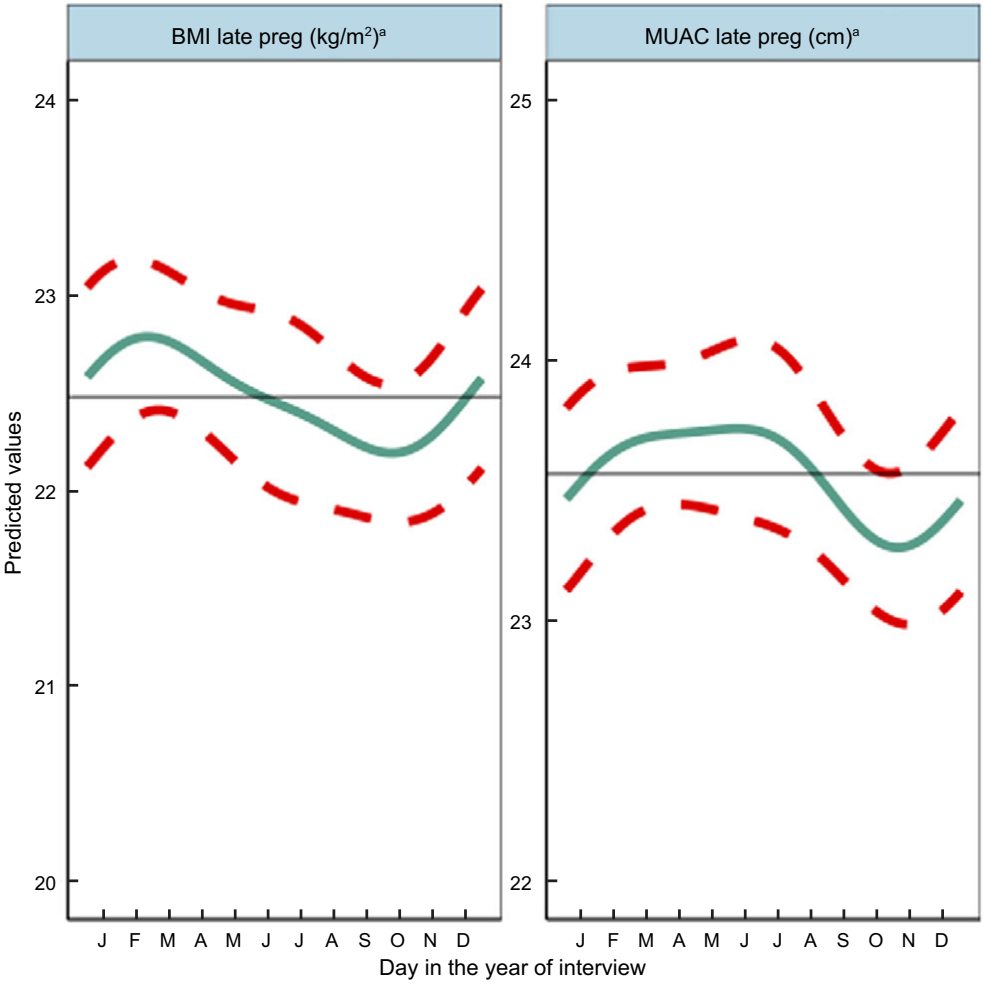
Predicted seasonal patterns of mothers’ BMI and MUAC in pregnancy plotted using cosinor analysis outputs in relation to day of measurement. a = significant annual cycle; s = significant semestral cycle; MUAC *n* 2816 and BMI *n* 2768; Note that the *y* axis range spans only 4 kg/m^2^ and 3 cm for BMI and MUAC, reflecting small yet statistically significant seasonal changes in maternal nutritional status

**Fig. 4:**
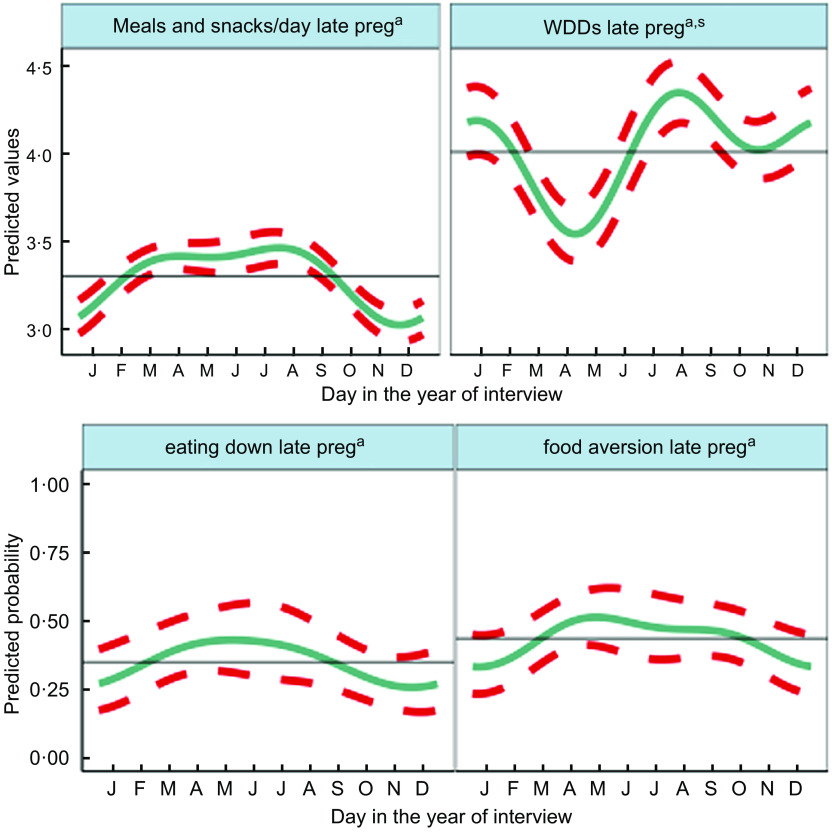
Predicted seasonal patterns of mothers’ number of eating occasions and Women’s Dietary Diversity Score (WDDS), eating less than when not pregnant and food aversion in pregnancy plotted using cosinor analysis outputs in relation to day of measurement. a = significant annual cycle; s = significant semestral cycle; *n* 2831 for each outcome

**Fig. 5:**
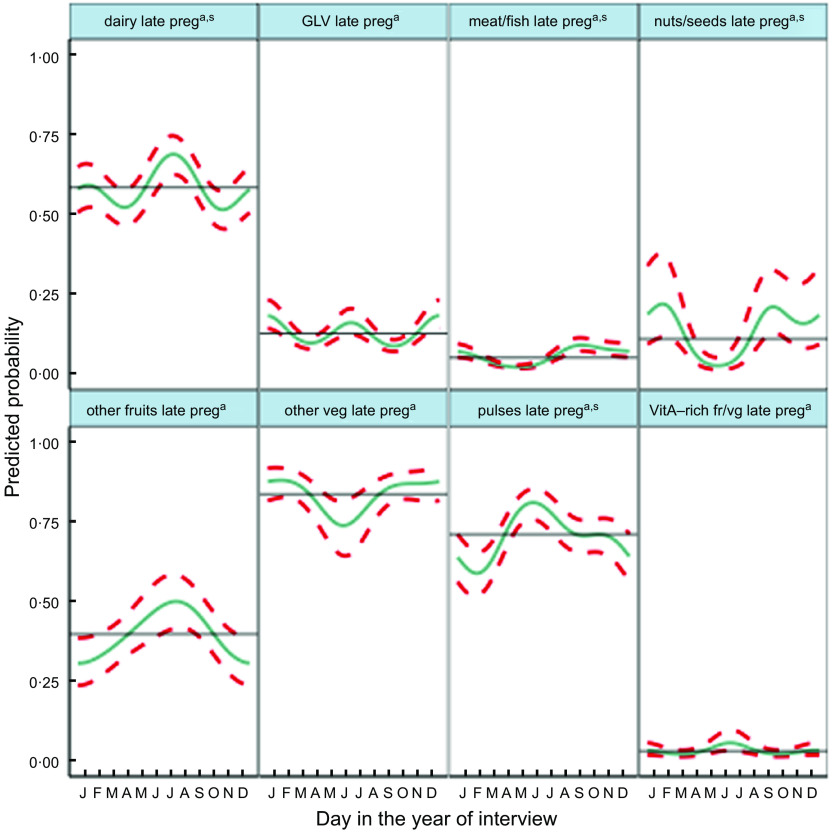
Predicted seasonal patterns of eight food groups included in the Women’s Dietary Diversity Score (WDDS) in pregnancy plotted using cosinor analysis outputs in relation to day of measurement. a = significant annual cycle; s = significant semestral cycle; *n* 2831

All fitted cosinor models showed evidence of significant clustering in the data, which required the use of random intercepts to resolve geographical clustering. Figs. 2–5 show predicted seasonal patterns for all relevant outcomes. Table 2 complements these plots, providing the relative size (amplitude), timing (acrophase) and statistical significance of the annual and semestral cycles of seasonality after adjustment for study arm, primigravida, gestational age and age of the infant. Table 2 also provides summaries over the whole sampling period including: mean newborn and pregnant mothers’ anthropometry, WDDS and meals; and probabilities of eating down, vomiting, food aversion, diarrhoea and food group consumption.

**Table 2:** Cosinor analysis results for annual and semestral (half-yearly) seasonality of newborn anthropometry and continuous and binary anthropometric and dietary outcomes in pregnancy

Infant z scores ≤72 h of birth	Mean	sd	Mesor	**Annual amplitude** ***(95 % CI)***		Annual Acro calendar day (95 % CI)		**Annual** ***P*** **-value**	**Semestral amplitude** ***(95 % CI)***		Semestral Acro calendar day (95 % CI)		**Semestral** ***P*** **-value**	***n***
LAZ	−1·051	1·080	−0·894	0·125	*(0·022, 0·227)*	***12-Dec***	***(30-Oct, 23-Jan)***	***0·0226***	0·087	*(0·012, 0·162)*	**15-Jun**	***(18-May, 12-Jul)***	***0·0324***	3291
WAZ	−1·122	0·997	−1·079	0·159	*(0·085, 0·233)*	***12-Jan***	***(23-Dec, 31-Jan)***	***<0·0001***	0·101	*(0·049, 0·153)*	**27-May**	***(8-Apr, 14-Jul)***	***0·0026***	3330
WLZ	−0·669	1·199	−0·367	0·411	*(0·283, 0·540)*	***1-Jan***	***(20-Dec, 12-Jan)***	***<0·0001***	0·103	*(0·030, 0·176)*	**11-Aug**	***(2-Jun, 19-Oct)***	***0·0096***	3029
HCAZ	−0·777	1·106	−0·857	0·046	*(*−*0·025, 0·117)*	*18-Dec*	*(19-Sep, 17-Mar)*	*0·6291*	*0·071*	*(0·007, 0·135)*	*19-Mar*	*(12-Mar, 25-Jul)*	*0·0863*	3200
**Pregnancy continuous outcomes**	Mean	sd	Mesor	Annual amplitude *(95 % CI)*		Annual Acro calendar day (95 % CI)		**Annual** ***P*** **-value**	Semestral amplitude *(95 % CI)*		Semestral Acro calendar day (95 % CI)		**Semestral** ***P*** **-value**	***n***
MUAC	23·70	2·08	23·11	0·223	*(0·019, 0·428)*	***5-Nov***	***(19-Aug, 21-Jan)***	***0·0132***	0·059	*(*−*0·046, 0·162)*	*5-Aug*	*(10-Jun, 28-Sep)*	*0·6447*	2816
BMI	22·65	2·48	19·05	0·271	*(0·102, 0·441)*	***15-Mar***	***(4-Nov, 23-Jul)***	***0·0001***	0·067	*(*−*0·064, 0·197)*	*28-Jul*	*(26-Jun, 28-Aug)*	*0·6845*	2768
WDDS	4·44	1·46	3·73	0·254	*(0·113, 0·394)*	***8-Oct***	***(28-May, 17-Feb)***	***<0·0001***	0·224	*(0·099, 0·349)*	**28-Jul**	***(26-Jun, 28-Aug)***	***<0·0001***	2681
Meals/day	3·45	0·92	3·03	0·196	*(0·073, 0·319)*	***11-Dec***	***(19-Nov, 1-Jan)***	***0·0001***	0·080	*(0·007, 0·154)*	*2-Jun*	*(20-Apr, 14-Jul)*	*0·0528*	2831
**Pregnancy binary outcomes**	Mean proportion	sd	Mesor (probability)	Annual amplitude in probability scale *(95 % CI)*		Annual Acro calendar day (95 % CI)		**Annual** ***P*** **-value**	Semestral amplitude in probability scale *(95 % CI)*		Semestral Acro calendar day *(95 % CI)*		**Semestral** ***P*** **-value**	***n***
Eating down	0·36	0·48	0·43	0·51	*(0·46, 0·57)*	***24-Dec***	***(23-Nov, 23-Jan)***	***0·0170***	0·47	*(0·43, 0·50)*	*15-Jul*	*(30-May, 29-Aug)*	*0·2005*	2831
Vomiting	0·09	0·28	0·07	0·09	*(0·06, 0·15)*	28-Jan	*(28-Oct, 29-Apr)*	*0·0608*	0·10	*(0·07, 0·15)*	*28-Jul*	*(8-Jun, 15-Sep)*	*0·0648*	2558
Diarrhoea	0·13	0·33	0·03	0·03	*(0·03, 0·04)*	8-Jan	*(24-Oct, 24-Mar)*	*0·4360*	0·03	*(0·03, 0·04)*	*15-Aug*	*(22-Jun, 7-Oct)*	*0·8282*	2831
Food aversion	0·29	0·45	0·35	0·44	*(0·37, 0·52)*	**30-Nov**	***(17-Oct, 12-Jan)***	***0·0048***	0·36	(0·33, 0·40)	*7-Jun*	*(16-Apr, 28-Jul)*	*0·8647*	2831
**Food group consumption**	Mean proportion	sd	Mesor (probability)	Annual amplitude in probability scale *(95 % CI)*		Annual Acro calendar day (95 % CI)		**Annual** ***P*** **-value**	Semestral amplitude in probability scale *(95 % CI)*		Semestral Acro calendar day (95 % CI)		**Semestral** ***P*** **-value**	***n***
Dairy	0·59	0·49	0·58	0·63	*(0·59, 0·68)*	**16-Jan**	***(4-Nov, 29-Mar)***	***0·0277***	0·64	*(0·61, 0·67)*	**22-Jul**	***(25-Jun, 17-Aug)***	***0·0011***	2831
Eggs	0·06	0·23	0·01	0·01	*(0·01, 0·02)*	29-Jan	*(2-Nov, 26-Apr)*	*0·0828*	0·01	*(0·01, 0·02)*	***17-Jul***	***(6-Jun, 26-Aug)***	***0·0480***	2831
GLV	0·36	0·48	0·12	0·14	*(0·11, 0·16)*	2-Feb	*(4-Nov, 2-May)*	*0·3617*	0·17	*(0·14, 0·20)*	**27-Jun**	***(10-Jun, 13-Jul)***	***<0·0001***	2831
Meat or fish	0·29	0·45	0·05	0·10	*(0·08, 0·12)*	***29-Oct***	***(11-Oct, 15-Nov)***	***<0·0001***	0·06	*(0·05, 0·07)*	**13-Aug**	***(3-Jun, 22-Oct)***	***0·0193***	2831
Nuts or seeds	0·27	0·45	0·11	0·25	*(0·15, 0·38)*	**28-Nov**	***(12-Nov, 13-Dec)***	***<0·0001***	0·18	*(0·14, 0·23)*	**28-May**	***(19-Apr, 5-Jul)***	***<0·0001***	2831
Other fruits	0·25	0·43	0·40	0·50	*(0·43, 0·56)*	**15-Jan**	***(30-Nov, 1-Mar)***	***0·0001***	0·41	*(0·37, 0·44)*	*27-May*	*(31-Mar, 22-Jul)*	*0·8976*	2831
Other veg’	0·82	0·38	0·84	0·89	*(0·85, 0·92)*	**13-Dec**	***(1-Nov, 23-Jan)***	***0·0040***	0·86	*(0·83, 0·88)*	*5-Jun*	*(16-Apr, 24-Jul)*	*0·3105*	2831
Pulses	0·71	0·45	0·71	0·79	*(0·75, 0·82)*	**7-Jan**	***(12-Dec, 1-Feb)***	***0·0001***	0·76	*(0·72, 0·79)*	**26-May**	***(30-Mar, 21-Jul)***	***0·0284***	2831
VitA fruit/veg	0·08	0·28	0·03	0·04	*(0·03, 0·06)*	25-Dec	*(26-Oct, 22-Feb)*	*0·2408*	0·04	*(0·03, 0·06)*	**23-Jun**	***(27-May, 19-Jul)***	***0·0371***	2831

GLV, green leafy vegetables; HCAZ, head circumference-for-age z-score; LAZ, length-for-age z-score; meals/day, includes meals and snacks per day; MUAC, mid-upper arm circumference; WAZ, weight-for-age z-score; WDDS, Women’s Dietary Diversity Score; WLZ, weight-for-length z-score; VitA fruit/veg, vitamin A-rich fruits and vegetables: eating down: eating less than when not pregnant; diarrhoea recall in last 2 weeks; food aversion includes loss of appetite.

Predicted plots of seasonal variability combine both the annual and semestral patterns into one curve. They refer to the timing of the peaks and nadirs by outcome, whilst *P*-values and amplitudes are taken from Table 2.

### Analysis of seasonality of neonatal anthropometry by month of birth

Table 2 shows the mean z-scores for neonatal LAZ, WAZ, WLZ and HCAZ. The percentages of newborns that were moderately and severely undernourished were: 12·3 % <−2 LAZ and 3·8 % <−3 LAZ (moderately and severely stunted, respectively); 11·8 % <−2 WAZ and 3·7 % <−3 WAZ (moderately and severely underweight, respectively); and 9·2 % <−2 WLZ and 3·2 % <−3 WLZ (moderately and severely wasted, respectively).

Figure 2 and Table 2 indicate significant annual and semestral cycles of seasonality for LAZ, WAZ and WLZ but not HCAZ at birth (adjusted for primigravida, age of the infant and study arm). The amplitude of the annual seasonal changes was larger (0·125–0·411 z-scores) than the semestral variation, which was close to 0·1 z-score but both are large enough to be of clinical significance. The annual nadirs and peaks calculated by adding or subtracting the amplitude to the mesor are: LAZ −1·019 to −0·770; WAZ −1·238 to −0·920; WLZ −0·778 to 0·045.

Children were born shorter but heavier in the winter (December/January), so had higher WLZ. Conversely, in the hot pre-/early-monsoon season, children had the lowest WLZ and WAZ (May/June), giving relatively high LAZ in July.

WLZ had the highest annual seasonal variation of 0·411 (95 % CI 0·283,0·540) z-scores (3–4 times greater than variability in WAZ or LAZ), with newborns least thin (higher WLZ) if born in the winter (January) and thinnest (lower WLZ) if born in the hot pre-monsoon season (May/June). The small yet significant semestral seasonality in WLZ was difficult to detect in plots. WAZ, like WLZ, was highest amongst those born in the winter (February) and lowest in those born in pre-monsoon (May/June) but the amplitude of seasonal WAZ variation was less than the variation in WLZ at 0·159 (95 % CI 0·085,0·233) z-scores. LAZ was the lowest (i.e. newborns were born shortest) in December, and 0·125 (95 % CI 0·022,0·227) higher in hot pre-monsoon and monsoon (March/April and July/August). A small dip in LAZ of 0·087 (95 % CI 0·012, 0·0162) amplitude was found during June, at the end of the hot pre-monsoon period (detected as a significant semestral cosinor term). HCAZ showed no significant seasonality in cosinor analysis, but in plots appeared very slightly lower in newborns born in the hot pre-monsoon (May) and higher in the winter (February) and monsoon (August).

### Seasonal patterns in pregnant mothers’ anthropometry

Results of adjusted cosinor analysis of mothers’ anthropometry by month of measurement are given in Fig. 3 and Table 2. Twenty-eight percent and 8·3 % were thin (MUAC 21–22·9 cm) and very thin (<21 cm), respectively. Mothers’ MUAC and BMI showed significant annual seasonality with amplitudes of 22 mm and 0·27 kg/m^2^, respectively, dipping to their lowest points of 22·9 cm and 18·78 kg/m^2^ in October during the pre-harvest period of highest food insecurity (Fig S4). BMI peaked in winter a few months after rice harvest (February/March) at 19·32 kg/m^2^, while MUAC gradually increased to a peak of 23·33 cm between March and June (pre-/early-monsoon) before falling over the monsoon months (July–September).

### Seasonal patterns in pregnancy diet

Figure 4 shows the combined annual and semestral predicted WDDS and meals and snacks (meals) per day and predicted probabilities of eating down and food aversion. WDDS showed larger and differing seasonal variation from other dietary outcomes. Meals per day varied by <0·5 between a trough in November during the busy food insecure harvest time, increasing over the winter to March and remaining high to peak in July/August (monsoon) when food security was moderate and agricultural (rice planting) workload heavy. Both eating down and food aversion were common (36 % and 29 %, respectively) and showed similar patterns to meals per day. They peaked in the hot/dry pre-monsoon season (April/May) and declined in the winter (November/December). Vomiting (9 %) and diarrhoea (13 %) in pregnancy showed no significant seasonality.

On average, mothers consumed 4·4 (SD 1·5) out of the 10 WDDS food groups in the preceding 24 h, one of which was invariably starchy staples including rice, wheat and potatoes. Cosinor results for WDDS showed significant seasonal variation with similar amplitude (of 0·22–0·25 food groups) in annual and semestral cycles. The cosinor plot showed the lowest WDDS in April during the extremely hot dry season, and the highest dietary diversity was in the late monsoon in August with a second peak in January and a smaller trough in October/November.

Consumption of food groups varied widely, except for starchy staples, consistently consumed daily year-round. The most commonly consumed groups in the preceding 24 h (Table 2 and Fig. 5) were other vegetables (82 %), pulses (71 %), dairy (59 %) and GLV (36 %). Nuts/seeds, meat/fish and other fruits were less frequently consumed in 27, 29 and 25 % of cases, respectively. Only 6 % of pregnant mothers ate eggs, while 8 % ate orange/yellow-coloured vitamin A-rich fruits and vegetables. Cosinor analysis of nine of the ten individual food groups that make up the WDDS (excluding starchy staples) (Table 2, Fig. 5) showed that seasonality varied widely by food group. Annual and semestral associations were found (in order of decreasing amplitude) for pulses, dairy, nuts/seeds and meat/fish. We found annual (but not semestral) seasonality for other vegetables and fruits, and semestral but not annual seasonality for GLV, vitamin A-rich fruits/vegetables and egg.

Plots in Fig. 5 demonstrate how seasonality varies by food group. Consumption of GLV had peaks in winter (January) and early monsoon (June) with troughs in April and September. Vitamin A-rich fruits and vegetables peaked in mango season in July with troughs in March and October and a much smaller peak in January. Meat/fish consumption was lowest in the hot season of May and highest at the time of the Dashain/Tihar festivals in September/October. Pulse consumption peaked in May/June after the April harvest, with the lowest consumption in the winter (February). Nuts/seeds and other vegetable consumption both dropped dramatically in May. Nuts/seeds consumption was the highest in February and September, whereas or other vegetables consumption stayed high between October and February. Conversely, other fruit consumption was highest in monsoon (July) and lowest in winter (December/January). Dairy consumption had a large peak in July and a smaller peak in January with troughs in April and October.

## Discussion

**Fig. 6:**
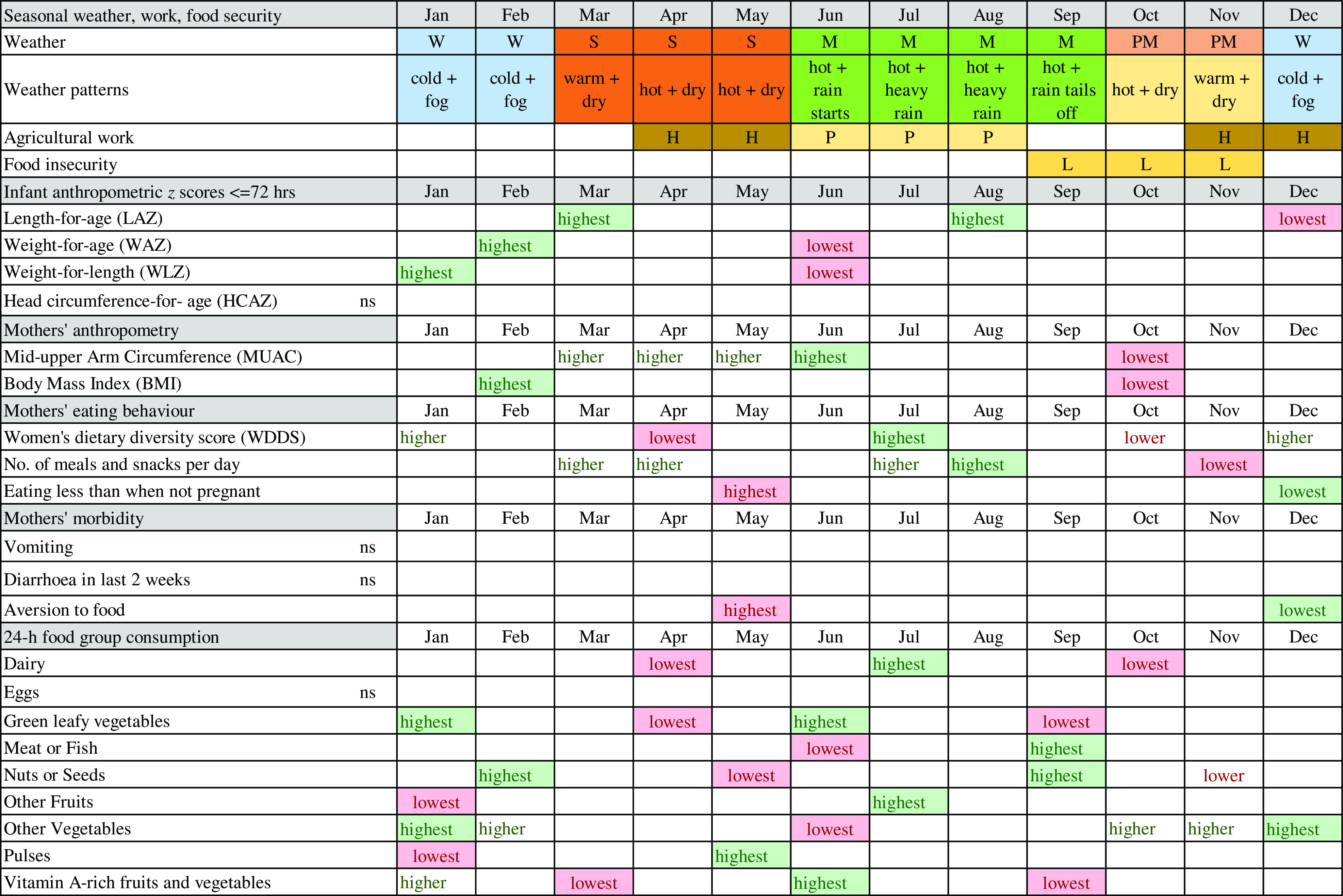
Schematic diagram of combined seasonal associations for pregnant mothers and neonates. Legend: lowest = major nadirs on plots; highest = major peaks on plots; ns = NS; green shading = positive effect on or indicator of nutrition; red shading = negative effect on or indicator of nutrition; W = winter; S = hot pre-monsoon; M = monsoon; PM =post-monsoon; H = harvest; P = rice planting; L = lean season

We found strong and differing seasonal patterns in maternal and newborn anthropometry and maternal food aversion, eating down, dietary diversity and consumption of individual micronutrient-rich foods in pregnancy, in particular, vegetables, dairy, pulses, fruits and nuts/seeds. Whilst our study was not designed to attribute causal associations between maternal and neonatal traits, any seasonality in foetal phenotype must be mediated by maternal biology. Hence, below we highlight potential factors that might explain these associations. Fig. 6 summarises the timing of major peaks and nadirs for outcomes with significant seasonality and seasonal patterns of weather, agriculture and food security to assist with the visualisation of these potential associations. In general, we observed that offspring seasonal associations track maternal associations with some time lag, which appeared to vary by neonatal outcome. These patterns are likely complicated by the fact that both ecological and cultural factors can contribute to seasonal changes in eating behaviour, food security and workload.

Seasonal variation in birth weight has been attributed to seasonality in food insecurity^(47)^, food intake, eating habits, workload and disease exposure (e.g. malaria)^(48)^ although malaria is uncommon in our setting^(49)^. In our study, food insecurity peaks before rice harvest (mid-September to mid-November), and pregnant mothers’ diets in October/November are low in vitamin A-rich fruits and vegetables, GLV and dairy. Conversely, the major festivals fall in these months with increased consumption of meat/fish, other vegetables and nuts/seeds. The long period of increasing food insecurity over the monsoon (July–September) which peaks in October, may lead to shortage of nutrients for foetal growth in pregnancy. Maximal foetal growth rates occur earlier in pregnancy for length than for weight^(50–52)^. Hence, being born short in the winter (December) may be associated with low MUAC and BMI of the mother some months earlier in October/November. Similarly, the LAZ peak in March occurs some months after the post-harvest improvements in food security and workloads. Poor maternal nutrition in the last trimester of pregnancy appears to be the likely cause of low birth weight in low-income settings^(53)^. Hughes et al found small for gestational age was the highest in Nepal in November, which coincides with the period of pre-harvest food insecurity^(54)^.

In winter, post-rice harvest, a combination of increased food security, lack of food aversion or heat stress, lower workloads and increased consumption of GLV, dairy and vitamin-A rich vegetables/fruits may be associated with rapid improvements in mothers’ energy and nutrient intake and faster weight gain of the infant *in utero*. This is consistent with BMI and MUAC peaks after the end of winter in February/March (and the sustained higher MUAC until June) and with newborns being least underweight and thin in winter (January/February). Foetal weight gain in late pregnancy tends to be due to fat accretion^(52,55)^, which might explain the winter WLZ peak. The February/March maternal peak in BMI and MUAC coincides with, or slightly lags behind, the peaks in WLZ and WAZ of the newborn (February). We speculate that additional maternal intake may be channeled to the newborn before maternal weight gain, or that gestational age at birth may be higher in winter, but further studies are needed to investigate this.

In the hot, early monsoon (June), mean WLZ and WAZ fell markedly (i.e. increased thinness) together with a small dip in LAZ. The pre-monsoon (March–May) is a time of heat stress^(10)^. High temperatures in pregnancy have been associated with low birth weight^(11,56–58)^ and preterm delivery^(59,60)^, hence the low WLZ and WAZ we detected in June could reflect more preterm deliveries. Other studies in South Asia found low birth weight and neonatal morbidity are higher^(61)^, newborns smaller^(54)^ and neonatal mortality higher^(8,54)^ in the summer/monsoon. In our study, despite relatively good food security in April/May, food aversion and eating down peaked, and dietary diversity was lowest, with troughs in consumption of meat/fish, GLV, vitamin A-rich fruits and vegetables, nuts and seeds, dairy and other vegetables. Only pulse consumption increased post-harvest in May. Pre-monsoon low availability of certain fruits and vegetables, short shelf-life and heat stress may contribute to decreased intakes, while harvesting of winter crops in April may increase energy expenditure^(23)^. These factors may limit foetal nutrient availability and lead to babies being born thinnest in June, consistent with falling pregnancy BMI (but not MUAC) over this time.

Birth size has been associated with micronutrient-rich food intake^(62–64)^, as has gestational weight gain^(65)^. In Bangladesh^(66)^ and Nepal^(28,67)^, studies found seasonal variation in pregnant mothers’ dietary diversity and food group consumption. Consistent with our findings, Campbell et al found the lowest meat/fish consumption in the hot season (May), and peaks of consumption of GLV in winter and mango in summer^(28)^. In Nepal, Jiang et al^(68)^ found seasonality in pregnant women’s intake of specific micronutrients which is likely to affect foetal growth and development considerably. Fasting in Ramadan might mimic the effect of seasonal changes in food intake upon pregnant women and has been hypothesised to be associated with lower birth weight. However, findings on this are inconsistent^(69,70)^ with both higher^(71)^ and lower^(72)^ placental weight reported in fasting women.

In Nepal, inequitable intra-household food allocation^(73,74)^ reduces the adequacy of pregnant mothers’ diets compared to their male household heads^(75)^. These inequalities may increase during seasons of shortage, especially for micronutrient-rich foods groups^(29)^ and during periods of peak agricultural activity^(30)^. The low status of young reproductive age women in our setting^(76–78)^ may magnify the impact of seasonal stresses, since ‘junior’ daughters-in-law usually cook and eat last, feeding micronutrient-rich foods to others while leaving little or none for themselves^(79)^.

Although we did not detect seasonality in recall of diarrhoea in pregnant women, other studies in Nepal have detected increased diarrhoea in the monsoon^(12,13)^ and in association with increased temperature^(80)^. Reduced appetite and increased demands on the body during illness may limit foetal growth and increase the risk of preterm delivery^(81,82)^. In rural India, higher maternal energy intakes, lower activity levels and exposure to winter (harvest) season predicted larger birth weight^(9)^. In Nepal, energy expenditure is highest during rice planting (pre-monsoon/monsoon) and lower in the winter^(23,24,83,84)^ and women gain weight when workloads decrease^(84)^. This is consistent with our finding that MUAC and BMI are low in October at the end of the monsoon and higher after the rice harvest (from March). A study in Bangladesh found, like us, that women were thinnest in October^(8)^.

The seasonal effects we have detected upon neonatal z-scores for LAZ, WAZ and WLZ are of clinical significance and are equivalent to effect sizes of public health interventions designed to improve newborn size such as lipid nutrient^(85)^ and iron folic acid^(86)^ supplementation of pregnant women. Lower LAZ at birth predicts linear growth retardation^(87)^. The seasonal changes in MUAC and BMI amount to 0·45 cm and 0·54 kg/m^2^ from nadir to peak, respectively. Though small, these differences in nutritional status of pregnant mothers are large enough to make a difference to newborn size at a population level.

Our findings may be helpful in the design of targeted pregnancy interventions. For example, the increased risk of newborn thinness and underweight in the hot season may call for pre-monsoon protein-energy supplements to be provided to pregnant women together with interventions to reduce heat stress and illness in this season. Similarly, supplements for pregnant women over the monsoon until rice harvest might be helpful in preventing maternal thinness in October and newborn stunting in the winter.

With increased frequency of extreme heat waves, flooding and drought in Nepal associated with climate change^(88,89)^, seasonal stresses and related diseases may increase over time^(80)^.

In general, our study confirms that Nepal has seasonal variation in the proximal determinants of undernutrition in the first 1000 d, as found in Madan et al’s review^(4)^. However, we provide information on seasonality of eating behaviours such as food aversion and ‘eating down’ in pregnancy, which have not been described elsewhere, and provide a more comprehensive view of seasonal variability in pregnancy diet and both maternal and newborn anthropometry.

Our large, detailed dataset on maternal and newborn anthropometry, maternal diets, eating behaviours and food security enables seasonal assessment of many outcomes within the same population, and provides insights into associated ecological and cultural factors.

Limitations include insufficient cases of linked newborn and maternal anthropometry/diet to conduct analyses of seasonal associations on both child and maternal outcomes within the same models, so we cannot infer causality. However, since all foetal environmental stimuli and stresses are transduced by maternal phenotype^(90)^, maternal exposures must in some way explain seasonality in newborn size. Low capture rates of enrolled women that resulted from premature stopping of data collection due to lack of funding, political unrest and migration of pregnant women to deliver at their parental home may have introduced biases, though these appear to be small^(91)^. Since our sample was from Province 2, findings may have limited external validity to the hills or mountains of Nepal but are likely to be generalisable to Maithili-speaking populations across Nepal and North India. There was a gap in data collection between 20 May and 7 July 2014 and some months had higher intensity of data collection than others. Nevertheless, we detected seasonal patterns in maternal behaviour, diet and nutritional status and neonatal anthropometry.

## Conclusions

Numerous components of maternal nutrition show seasonality in lowland Nepal, and this pattern extends to newborn size. Seasonality should therefore be accounted for when designing, implementing and evaluating public heath interventions to prevent maternal and child undernutrition in South Asia. Studies should collect data across different seasons and adjust for seasonality in analyses. Interventions could also deliberately target windows of seasonal vulnerability to wasting and anaemia. For longitudinal studies where data collection across the seasons cannot be arranged, data should be collected in the same season at each round, to avoid misinterpretation of apparent time trends that might be attributable to differing season of measurement.

## Supplementary material

For supplementary material accompanying this paper visit https://doi.org/10.1017/S1368980021003633. click here to view supplementary material
