# Supplementary material for: Comprehensive analysis of the association of seasonal variability with maternal and neonatal nutrition in lowland Nepal
Source: Public Health Nutr. 2021 Aug 23;25(7):1877–92. doi: 10.1017/S1368980021003633 (PMC9991647; doi:10.1017/S1368980021003633)
Supplement: Supplementary file 1 [file S1368980021003633sup001.zip › S1368980021003633sup001/S1368980021003633sup001.docx]

**S3 Fig. Number of cases per day of measurement for late pregnancy anthropometry and by day of birth for newborn anthropometry**

**
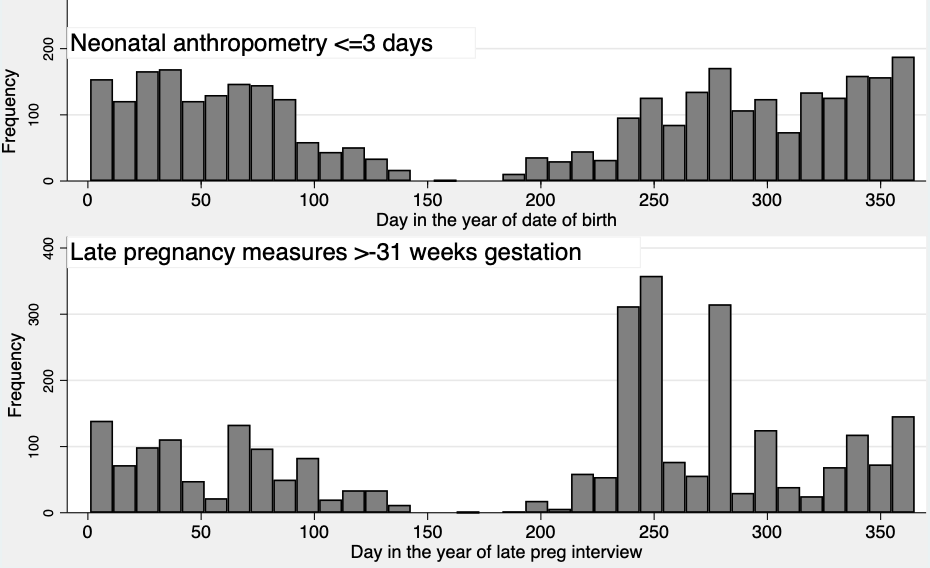
**

late pregnancy *n*=2,831; birth anthropometry ≤72 hours *n*=3,330.
