# Supplementary material for: Comprehensive analysis of the association of seasonal variability with maternal and neonatal nutrition in lowland Nepal
Source: Public Health Nutr. 2021 Aug 23;25(7):1877–92. doi: 10.1017/S1368980021003633 (PMC9991647; doi:10.1017/S1368980021003633)
Supplement: Supplementary file 1 [file S1368980021003633sup001.zip › S1368980021003633sup001/S1368980021003633sup002.docx]

**S5 Annex. Cosinor analysis model specifications of annual and semestral (half-yearly models) for continuous and binary outcomes**

Full specification of the fitted cosinor model with both annual and semestral terms for a continuous outcome $Y_{ij}\left( t \right),$ measured on mother (or child) *i* belonging to cluster *j* at time *t* (in days):

$Y_{ij}\left( t \right)=M_{j}+\beta_{1} \sin\left( \frac{2\pi t}{D} \right){+ \beta}_{2} \cos\left( \frac{2\pi t}{D} \right)+\beta_{3} \sin\left( \frac{4\pi t}{D} \right){+ \beta}_{4} \cos\left( \frac{4\pi t}{D} \right) + {\gamma^{T} X+ \varepsilon}_{ijt}$,

The semestral acrophase $\phi'$ can be converted to days (from the start of the cycle) as follows:

$$Acrodegrees for semestral seasonality=\left( \frac{(- \phi'*360)}{4\pi} \right)$$

$$Acrocalendar days for semestral seasonality=\left( \frac{(- \phi'*365)}{4\pi} \right)$$

The equivalent model for a binary outcome $Z_{ij}\left( t \right),$ measured in mother (or child) $i$ belonging to cluster *j* at time *t* (in days) is:

$${g(Z}_{ij}\left( t \right))=M_{j}+\beta_{1} \sin\left( \frac{2\pi t}{D} \right){+ \beta}_{2} \cos\left( \frac{2\pi t}{D} \right)+\beta_{3} \sin\left( \frac{4\pi t}{D} \right){+ \beta}_{4} \cos\left( \frac{4\pi t}{D} \right) + \gamma^{T} X= LP$$

where $g(\cdot)$ is the logit link function and $LP$ is the linear predictor. The model’s coefficients are fitted by maximum likelihood and probabilities can be predicted for values of angles (dates) $t$ and the covariates $X$ in the $LP$ using the inverse logistic transformation:

$pr=\frac{e^{LP}}{1+e^{LP}}$.

For the binary outcomes we then express amplitude and mesor on the probability scale as follows:

$$\mathrm{Mesor}\left( on probability scale \right)=\frac{e^{M_{j}}}{1+e^{M_{j}}}$$

$$Amplitude (on probability scale)=\frac{e^{M_{j}+amplitude}}{1+e^{M_{j}+amplitude}}$$
