# Supplementary material for: Comprehensive analysis of the association of seasonal variability with maternal and neonatal nutrition in lowland Nepal
Source: Public Health Nutr. 2021 Aug 23;25(7):1877–92. doi: 10.1017/S1368980021003633 (PMC9991647; doi:10.1017/S1368980021003633)
Supplement: Supplementary file 1 [file S1368980021003633sup001.zip › S1368980021003633sup001/S1368980021003633sup003.docx]

**S2 Fig. Participant flow of women enrolled in the Low Birth Weight South Asia Trial and follow-up rates in late pregnancy and after delivery**

NB: If a data collector had a high proportion of outlying Length-for-Age or Weight-for-Age Z scores at the <72 hour time point, then all cases for these data collectors were dropped due to providing “questionable quality data” (n=114). Out 73 data collectors, six had <6, four had 9 to16 and one had 43 measures excluded respectively.
