# Supplementary material for: Comprehensive analysis of the association of seasonal variability with maternal and neonatal nutrition in lowland Nepal
Source: Public Health Nutr. 2021 Aug 23;25(7):1877–92. doi: 10.1017/S1368980021003633 (PMC9991647; doi:10.1017/S1368980021003633)
Supplement: Supplementary file 1 [file S1368980021003633sup001.zip › S1368980021003633sup001/S1368980021003633sup004.docx]

**S1 Fig. Seasonal weather patterns in Janakpur, Nepal with agricultural activities and lean season indicated.**


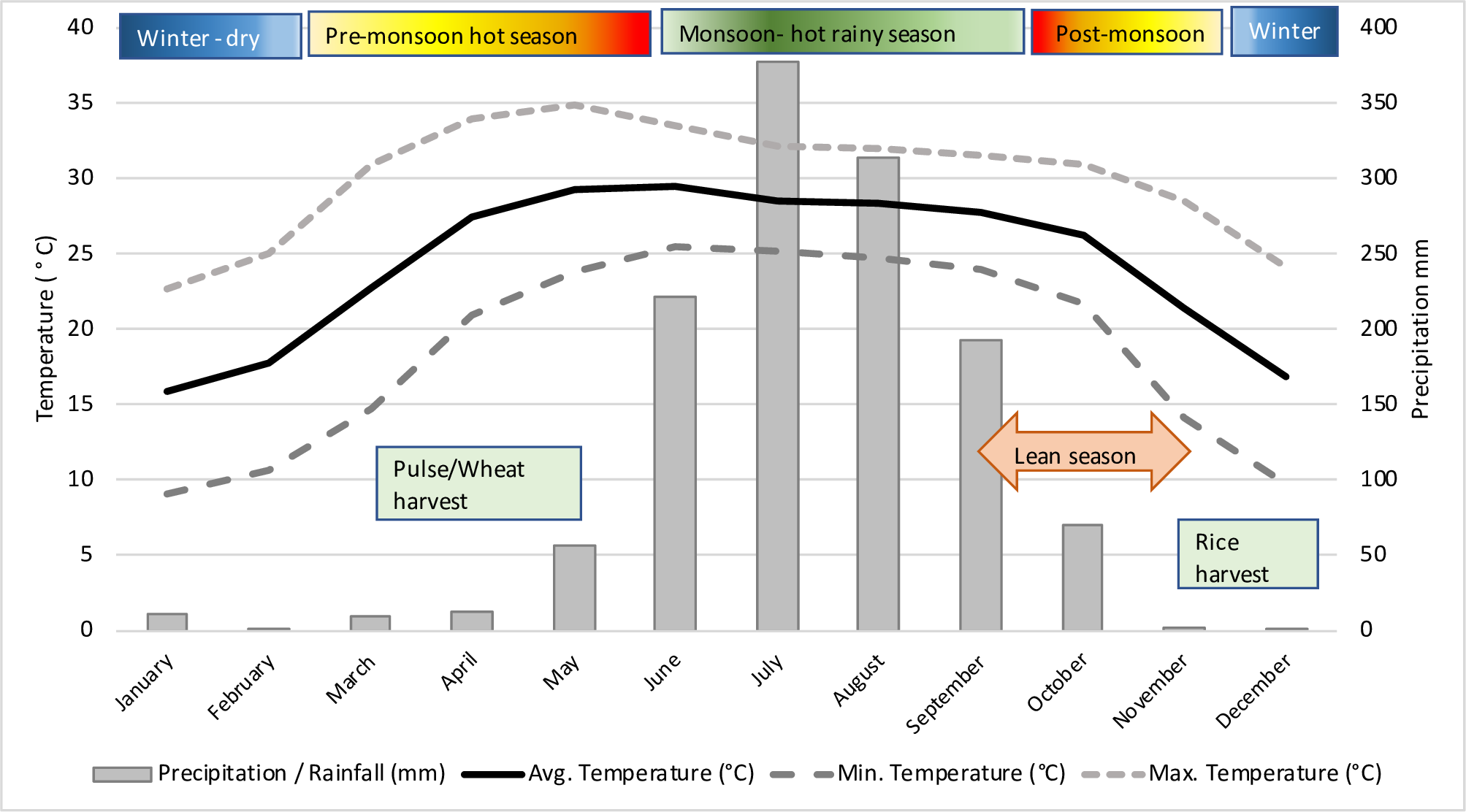


Produced using data from <https://en.climate-data.org/asia/nepal/central-development-region/janakpur-51372/> Accessed 3 June 2019.
