# Supplementary material for: Comprehensive analysis of the association of seasonal variability with maternal and neonatal nutrition in lowland Nepal
Source: Public Health Nutr. 2021 Aug 23;25(7):1877–92. doi: 10.1017/S1368980021003633 (PMC9991647; doi:10.1017/S1368980021003633)
Supplement: Supplementary file 1 [file S1368980021003633sup001.zip › S1368980021003633sup001/S1368980021003633sup005.docx]

**S4 Fig. Seasonality of Nepalese months^a^ of food insecurity as reported by mothers in early pregnancy.**

**
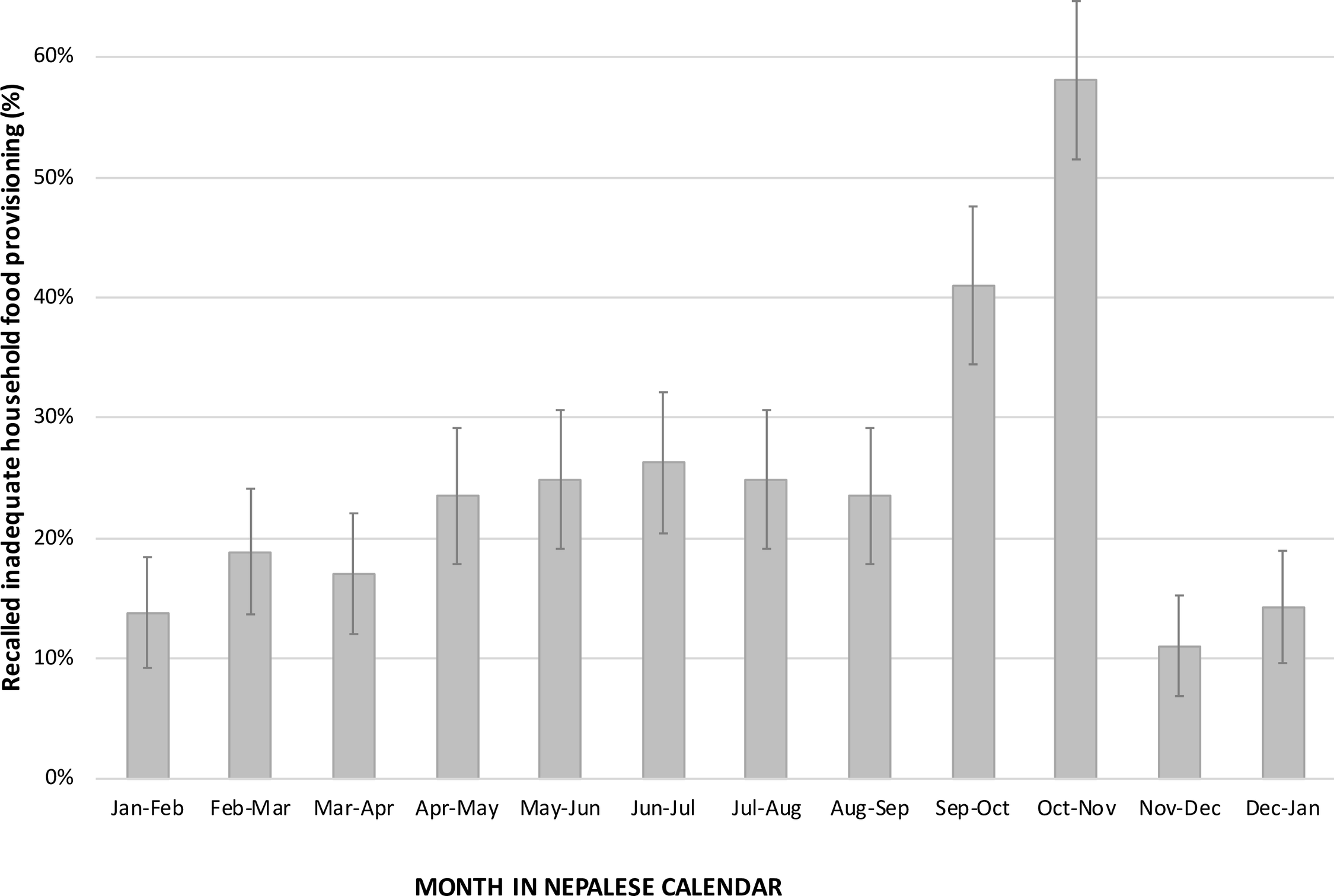
**

*n*=217 mothers who said they experienced at least one month of inadequate household food provisioning in the year preceding the pregnancy interview (i.e. recalling the months between 27 Jan 2013 and 31 Mar 2014). Error bars indicate 95% confidence interval. ^a^ As most rural Nepalese respondents are not familiar with Gregorian months, recall of which months had food shortages was in Nepalese months. These are offset from Gregorian months by approximately 15 days.
